# Genome integrity, stem cells and hyaluronan

**DOI:** 10.18632/aging.100438

**Published:** 2012-02-26

**Authors:** Zbigniew Darzynkiewicz, Endre A. Balazs

**Affiliations:** ^1^ Brander Cancer Research Institute & Department of Pathology, New York Medical College, Valhalla, NY 10595, USA; ^2^ Matrix Biology Institute, 725 River Road, Suite 205, Edgewater, NJ 07020, USA

**Keywords:** Hyaluronic acid, stem cells, stem cells niche, germinal cells, sperm cells, oocytes, oxidative DNA damage, reactive oxidative species (ROS), inflammation, cell cycle, apoptosis

## Abstract

Faithful preservation of genome integrity is the critical mission of stem cells as well as of germ cells. Reviewed are the following mechanisms involved in protecting DNA in these cells: (a) The efflux machinery that can pump out variety of genotoxins in ATP-dependent manner; (b) the mechanisms maintaining minimal metabolic activity which reduces generation of reactive oxidants, by-products of aerobic respiration; (c) the role of hypoxic niche of stem cells providing a gradient of variable oxygen tension; (d) (e) the presence of hyaluronan (HA) and HA receptors on stem cells and in the niche; (f) the role of HA in protecting DNA from oxidative damage; (g) the specific function of HA in protecting DNA in stem cells; (h) the interactions of HA with sperm cells and oocytes that also may shield their DNA from oxidative damage, and (e) mechanisms by which HA exerts the anti-oxidant activity. While HA has multitude of functions its anti-oxidant capabilities are often overlooked but may be of significance in preservation of integrity of stem and germ cells genome.

## Stem cells: keeping genotoxins out of the cell

Faithful preservation of genome integrity throughout lifetime of the organism is the critical mission of the long-term self-renewal stem cells. Several mechanisms, both intrinsic to stem cells themselves as well as extrinsic, provided by the microenvironment, (stem cell niche) serve this purpose. One of the intrinsic mechanisms is aimed to effectively exclude potentially hazardous agents that enter the cell from outside. This activity is mediated by high level of expression of multidrug-resistance gene (*MDR1*)-encoded adenosine triphosphate-binding cassette (ABC) transporter P-glycoprotein (P-gp). This efflux machinery can pump out variety of genotoxins in ATP-dependent manner [1]. There are over 30 members of ABC transporter super-family genes, whose protein transcripts are able to remove wide range of substrates out of the cell [2-4]. The degree of efflux activity correlates with differentiation; namely the maximal activity are expressing the most primitive long-term self-renewal stem cells [5]. Another possible function for these efflux pumps is the removal of small lipophilic regulatory molecules such as steroids, whose presence may activate growth or differentiation [6].

It should be noted that the exceptionally high activity of the efflux pump provides a useful marker to identify and isolate (sort out) stem and early progenitor cells. This is being achieved using fluorescent efflux substrates such as Hoechst 33342 [5, 7, 8] or rhodamine 123 [9, 10] in conjunction with flow cytometry and electronic cell sorting. Since retention of these fluorochromes in stem cells is impeded due to their rapid efflux, attributed primarily to *MDR1* activity [11], stem cells can be recognized (and sorted out) as a distinct cell subpopulation characterized by much reduced fluorescence intensity. In the case of staining with Hoechst 33342 the hematopoietic stem cells are characterized by the reduced intensity of fluorescence combined with a metachromatic shift of this fluorochrome, revealed as so called “side population”, having much distinct fluorescence emission properties compared to other bone marrow cells [5, 8].

## Oxygen danger

One of the strong genotoxins is oxygen [12, 13]. Oxidative DNA damage leads to oxidation of DNA bases with predominance of guanine [formation of 8-oxo-7,8-dihydro-2'-deoxyguanosine (8-oxo-dG)], base ring fragmentation, sugar modification, crosslinking of DNA and protein as well as induction of DNA strand breaks [14, 15]. The most deleterious effect of oxidative DNA damage is formation of DNA double-strand breaks (DSBs). These lesions can be repaired either by recombinatorial repair or non-homologous DNA-end joining (NHEJ). The template-assisted recombinatorial repair is essentially error-free but can take place when cells have already replicated their DNA which serves as a template, i.e. in late-S and G_2_ phase of the cell cycle. DNA repair in cells lacking a template (G_1_ and early S phase) occurs by the NHEJ mechanism. The latter however, is error-prone and may result in deletion of some base pairs [16]. When such change is at the site of an oncogene or tumor suppressor gene it may promote tumorigenesis [17]. It can also cause translocations and telomere fusion [18]. When the stem cells reside in the in the non-proliferating G_0_ state the error-prone NHEJ is the only mechanism for their DSBs repair.

Both, the exogenous oxygen as well as reactive oxygen species (ROS), the by-products of aerobic respiration within the cell, contribute to oxidative DNA damage [19-20]. The primary ROS generated in mitochondria can diffuse from these organelles and reach the nuclear DNA inducing its damage, or can generate secondary radicals with DNA damaging properties [21, 22]. The ongoing oxidative DNA damage by endogenous oxidants induces consistent replication stress, which when concurrent with activation of mTOR pathways, is considered to be the primary cause of cell senescence and organismal aging [23-28]. Clearly, the genome of stem cells has to be maximally protected from senescing- and aging-related changes. What mechanisms operate to shield genomic DNA in stem cells from the oxidative damage?

## Reduced oxygen level in the environment: the hypoxic niche

One of the factors reducing oxidative DNA damage in stem cells is their location. Stem cells reside in their respective niches [29], the distinct anatomical compartments composed of cellular and intercellular matrix constituents providing an optimal milieu for maintenance and regulation of their biologic processes [30]. The very characteristic feature of stem cell niche is low concentration of oxygen [31, 32]. While percentage of oxygen in ambient air is about 21% [partial pressure, oxygen tension p(O_2_) = 159 mm Hg], its percentage in most organs and tissues is reduced to 2% – 9% (15 – 68 mm Hg) (33,34) and in the environment of stem cell niche is between 1% and 6% (7.6 – 45 mm Hg) and the intracellular p(O_2_) is 3 – 4 mm Hg [35, 36].

Interestingly, at the very low oxygen tension (1%) the stem cells remain in the non-proliferating compartment and maintain pluripotency while at the increased tension (3% – 5%) they proliferate and start differentiation [36, 37]. The oxygen tension gradient within the stem cell niche appears to provide signaling that mediates transition of these cells from quiescence to proliferation and trigger their differentiation [38-42]. At the same time the very low oxygen tension milieu, where the long-term self-renewal stem cells reside, offers the conditions in which oxidative DNA damage induced by exogenous oxygen is much reduced. Based on differences in p(O_2_) such damage is expected to be nearly 20-fold lower in the stem cell niche than in tissue culture maintained at ambient air.

## Containing danger of endogenous oxidants

Several mechanisms are being used by stem cells to mitigate the hazards conveyed by the endogenous reactive oxidants. First of all, for most of their life stem cells remain in quiescent state having minimal metabolic rate and thereby low level of ROS production [43-47]. Their quiescent state is comparable to that of the peripheral blood lymphocytes, whose metabolic rate and generation of oxidants is nearly two orders of magnitude lower that of their mitogenically stimulated counterparts [48, 49]. The metabolic rate of the most primitive stem cells, the “very small embryonic-epiblast stem cells”(VSELs), as judged from extreme paucity of their cytoplasmic content and few mitochondria, is expected to be indeed minimal [50].

Further mechanism designed to maintain minimal metabolic rate may involve asymmetric cell division. It was recently shown that the most primitive, long-term reconstituting, hematopoietic stem cells (Lin^−^Sca^+^Kit^+^; LSK) which continuously reside in the LSK compartment, during division segregate their mitochondria unequally delegating more of these organelles to that of the daughter cell which exits the LSK compartment and enters differentiation pathway. The sister cell remaining in the LSK compartment inherits the very low level of energized mitochondria and thus remains with minimal level of ROS production [51, 52].

## Hyaluronan in stem cells biology

Hyaluronan (hyaluronic acid; HA), the ubiquitous component of intercellular matrix, carries out numerous functions that are essential for survival, differentiation, proliferation, motility and intercellular communication of variety of cell types. HA is also of crucial importance for tissue and organ development and architecture [reviews in 53]. Depending on its molecular weight HA may exert diverse effects on normal and cancer cells functions [53-57]. Extensive evidence points out that HA plays critical role in many facets of stem cells biology. Most of the evidence comes from the studies of Susan Nilsson and her collaborators of the Peter MacCallum Cancer Center, Melbourne, who explored the interactions of this glycosaminoglycan with human and murine hematopoietic stem cells [55-59]. These authors demonstrated that HA is being synthesized by HA-synthases located in the plasma membrane of stem cells and is expressed on the surface of these cells [44, 56]. Three HA synthases, HAS-1, HAS-2 and HAS-3, each synthesizing this biopolymer at different molecular weight, are involved in its production in stem cells. HA is also a predominant component of intercellular matrix of the stem cell niche. It is unclear to what extent stem cells themselves and the stromal cells (fibroblasts) respectively contribute to its accumulation in the niche [59, 60].

The level of expression of HA on the cells surface correlates with the differentiation status of the stem and progenitor cells. The highest HA expression is observed on the surface of the most primitive (Lin^−^) stem cells, and a progressive decline in its expression is seen to be concurrent with differentiation. The mechanistic *in vitro* studies indicate that HA functions in modulating cell proclivity to differentiation and proliferation by enforcing continuance of the dormant state of the most primitive Lin^−^ cells [55-61]. This inhibitory effect of HA is consistent with its suppressive effect on mitogenic stimulation of lymphocytes which also remain in the G_0_ dormant state when cultured with the mitogen in the presence of high molecular weight HA [62].

HA plays also essential role in homing and lodgment of the transplanted stem cells. The transplanted hematopoietic stem and progenitor cells preferentially home to the trabecule-rich metaphysic of femur where they become lodged in endosteal niche, being associated with blood vessels. The presence of HA, which is highly expressed in endothelium of blood vessels, provides the homing and lodgment mechanism for the stem cells having strong expression of the HA receptors CD44 and RHAMM [57, 63, 64]. It appears that HA synthase HAS-3 plays a major role in this mechanisms because transplantation of stem cells to mice lacking this synthase (Has-3^−/−^) leads to aberrant distribution of the grafted cells in bone marrow compared to the wild type recipients [58]. Apparently the presence of active HAS-1 and HAS-2 synthases is inadequate to assure proper homing and lodging of hematopoietic stem cells in Has-3^−/−^ mice. The presence of HA CD44 receptors on surface of stem cells mediates their adhesion and rolling movement on HA surfaces [65].

One of the mechanisms by which HA facilitates homing and engraftment of transplanted hematopoietic stem cells (as shown in the case of umbilical cord blood) involves its effect in promoting synthesis of membrane type 1 (MT1) of matrix metalloproteinase 2 (MMP-2) [66]. Since MT1-MMP-2 plays an important role in homing of hematopoietic progenitor stem cells it has been proposed that the priming strategy that involves pretreatment of cord blood progenitor/stem cells with HA before transplantation could improve their homing and engraftment [66].

Interestingly, HA can substitute hypoxia for the long-term maintenance of embryonic stem cells in culture, preserving their pluripotency and thereby providing a useful alternative that enhances their viability [67]. A combination of hypoxia and HA may be even more beneficial in this application.

The importance and protective effect of HA on stem cells are well recognized in the field of transplantation of these cells in regenerative medicine. Variety of hydrogel scaffolds providing a niche-like environment, are being used as stem cell vehicles for their transplantation [68-73]. HA, modified by different approaches to allow structural encapsulation of individual stem cells, is the key component of all these hydrogel products. Stem or progenitor cells encapsulated into such bio-artificial niches have been found to be protected from cytotoxic agents such as anticancer drugs used to treat the patient, and remain competent in terms of their capability to lodge and functionally replace native stem cells [69-73].

It should be noted that the HA receptor CD44 is frequently used as a marker to identify and sort out drug-resistant stem-like cells from different tumors [74-76]. Interactions between HA and cancer cells are the subject of extensive literature [reviews in 77] but little is known on the role of HA and HA-CD44 interactions in cancer stem-like cells, particularly whether such interactions may affect stability of these cells genome. However, there are interesting observations from the studies of cancer cells pertaining to interactions between CD44 and HA vis-à-vis the efflux pump in these cells affecting efficiency of the P-glycoprotein in removing anticancer drugs of known genotoxicity [78-81]. It is possible that the HA-CD44 interactions in cell membrane of stem cells play a role in protecting their genome via modulation of the efflux pump effectiveness.

## Protection of DNA from oxidative damage by hyaluronan

The increased production of ROS takes place during inflammation and the oxidants generated in the inflamed tissue mediate and further amplify many inflammatory reactions [82-87]. It has been proposed that one of HA biological functions is to provide protection against cellular damage caused by radicals generated by oxidative reductive systems or ionizing radiation [88]. Indeed, HA has strong anti-inflammatory properties and is clinically used to abrogate or attenuate inflammation in many tissues and organs [89-100]. Its clinical utility is of particular significance in treatment of osteoarthritis [89, 95-97, 100]. The anti-inflammatory capabilities of HA are mediated, at least in large part, by its antioxidant activity [101-108]. Promotion of the wound healing by HA may also be facilitated by similar antioxidant mechanisms [109, 110].

We have recently reported that DNA damage signaling evoked by exposure of cells to exogenous oxidants such as H_2_O_2_ was markedly attenuated by HA; especially effective was HA of high molecular weight [111]. In another study, we observed that the extent of DNA damage response induced during oxidative burst of macrophages [112] was also distinctly reduced in the presence of high molecular weight HA [113]. Moreover, the level of constitutive DNA damage signaling revealed by activation of *Ataxia Telangiectasia Mutated* (ATM) and phosphorylation of histone H2AX on Ser139 and reporting persistent DNA damage induced by endogenous oxidants, the by-products of aerobic respiration in mitochondria [114, 115], was seen to be strongly attenuated by HA [111]. Here again, the high molecular weight HA was more effective than its low molecular weight form [113]. These findings underscore that HA exerts a protective effect on cellular genome by neutralizing the exogenous as well as endogenous oxidants.

Protection of DNA by HA through its antioxidant activity has been also observed in fibroblast cultures in which oxidative stress was induced by treatment with iron and ascorbate [116]. In addition to protection of DNA integrity (assessed by analysis of its fragmentation) HA also reduced protein oxidation, lipid peroxidation and formation of OH* radicals in these cultures [116].

## Does hyaluronan also protect germ cells DNA?

Interestingly, HA has found wide utility in assessment of integrity of sperm cells DNA. Specifically one of the methods to separate fertile sperm cells that have undamaged DNA is based on the ability of sperm cells attach to HA [117-121]. The sperm cells that bind to solid-state HA show chromatin structure with high DNA chain integrity [116-120]. DNA integrity of HA-bound sperm cells was comparable to that assessed by the alternative methods of spermatozoa evaluation, based on DNA susceptibility to denaturation [122] or analysis of the presence of DNA strand breaks by the TUNEL assay [123]. Sperm cells express HA-receptors on the cell surface and it has been postulated that the HA-binding receptors have a specific role in cell maturation, motility and fertilization processes [124-126]. Since the cells expressing HA-receptors can internalize HA by endocytosis [127-129] it is possible that HA is internalized into spermatozoa and its presence within the cell can provide further DNA protection from oxidative damage. Since in sperm cells the ROS-generating organelles mitochondria are at some distance from nuclear DNA the HA-protection from exogenous rather than endogenous oxidants may be of more significance.

Oocytes were shown also to synthesize and secrete HA [130-133]. The concentration of HA in follicular fluid was shown to a good indicator for estimation of oocyte viability for fertilization [130]. The interactions between HA and CD44 receptors on cumulus-oocyte complexes were shown to be critical for oocyte maturation [133, 134].

Similar to stem cells there are several mechanisms protecting DNA in germ cells, including the blood-testis barrier and efflux pumps [135-137]. The possible role of HA in terms of protection of germinal cells DNA against oxidative damage has not been addressed in the literature as yet. However, the collective evidence of strong correlation between the expression of HA-receptors versus genome integrity of sperm cells [119-122] as well as versus viability and capability of oocytes for fertilization [132], combined with the findings that HA protects DNA from oxidative damage in other cell types [111-113, 116], provides a contention that HA may play a role in protecting germ cells genome from oxidative damage.

## Mechanisms of cell protection from oxidative damage by HA

As discussed, strong evidence points out that the antioxidant properties of HA provide protection of genomic DNA against the damage by ROS as well as are involved in attenuation of inflammatory processes. Two different mechanisms may contribute to the antioxidant properties of HA. One mechanism involves the ability of HA to chelate Fe++ and Cu++ [138, 139]. These ions are critical in Fenton's reaction, in which the superoxide and hydrogen peroxide, which themselves are not highly reactive with DNA, are converted into the strongly reactive with DNA hydroxyl radical (OH*) [140-142]. Interception of these ions by HA makes them unavailable for Fenton's reaction thereby reducing generation of the OH* radical.

The second mechanism by which HA may attenuate damage induced by oxidants involves the direct scavenging of oxidant molecules, particularly the reactive products of Fenton's reaction OH* radicals, by HA [101, 103, 116]. Of note, HA from synovial fluid was shown to have greater ROS-removing activity and scavenged more diverse range of ROS compared to other antioxidants such as catalase or superoxide dismutase (SOD) [103].

The ROS scavenging by HA while it depletes the pool of reactive oxidants potentially damaging DNA at the same time results in a breakdown of HA molecules [101, 103, 104]. The loss of HA viscosity, reporting HA degradation, is observed *in vitro* upon HA exposure to free radicals and in *vivo*, in the inflamed tissues [143]. It was shown that exposure of synovial fibroblasts to H_2_O_2_ while leads to HA breakage it also enhances HA synthesis [144]. Likely, this is a compensatory mechanism to maintain high level of HA thereby enhancing the antioxidant defense [145]. Because the newly synthesized HA is of high molecular weight and is replacing the degraded HA the capability of ROS scavenging is being preserved and not decreased.

When DNA damage occurs the highly effective and as much as possible error-free DNA repair is the subsequent mechanism by which stem cells protect genome integrity. Robustness of DNA repair in stem cells, and in particular of embryonic stem cells, is greater compared to other somatic cells [145-148]. For example following DNA damage mouse embryonic stem cells end up with 100 times fewer cells with mutations compared to mouse adult somatic or embryonic fibroblasts [145]. One mechanisms contributing to such an outcome is modulation of the cell cycle checkpoints and DNA damage signaling pathways to enhance efficiency of DNA repair. The second mechanism relies on elimination of cells with mutations by apoptosis. The checkpoint and DNA damage signaling pathways as well as the pathways regulating cell proclivity to undergo apoptosis are much more effective in stem cells compared to other somatic cells [145].

## Possible pitfalls in interpretation of the data from *in vitro* experiments

Most observations regarding modulation of cell growth and proliferation of stem cells by HA came from the *in vitro* experiments in which the cells were growing at standard tissue culture conditions in an atmosphere of air and CO_2_. As mentioned, the partial pressure of O_2_ (pO_2_) of 159 mm Hg (21%) in ambient air is much higher than in tissues, especially in the microenvironment of the stem cells niche [35, 36]. Because cell growth, and in particular the induction of proliferative senescence [149] as well as accumulation of mutations [150], are much dependant on oxygen concentration, the *in vitro* findings on cells growing under standard atmosphere conditions may be hampered by an experimental bias, i.e., the consequence of exposure to non-physiological pO_2_.

Most of the *in vitro* studies were also carried on stem cells growing attached to glass or plastic surfaces and exposed to HA that was included in the culture media. Such cells when attached to solid, dried out surfaces treated with various chemicals have very different migration properties, proliferation capability, and cell-to-cell interactions compared to *in vivo* conditions. The cells growing on solid surfaces exhibit also a very different behavior than that of the same cells grown on highly viscoelastic HA.

When cells from the lymphomyeloid system, tumor cells, fibrocytes or stem cells are placed on highly hydrated elastoviscous solutions of HA, they remain round, divide and move toward each other and on top of each other, forming a multilayered assembly. These assemblies are separated from each other. Depending on the cell lines, they survive or die slowly [53, 62, 103, 151, 152]. This is the same highly purified hyaluronan that today is used worldwide as a therapeutic device in eye surgery, replacing the synovial fluid to control pain and as tissue augmentation of connective tissues of the skin and muscles.

Since in the vertebrate intercellular matrix, especially in the connective tissues, the cells are surrounded by liquid hyaluronan of various molecular weights and concentrations, it is logical to argue that the cells must have different behavior and activities – even gene expression – when surrounded with hyaluronan and not growing on plastic or glass surfaces. A caution, therefore, should be exercised in interpolating the *in vitro* results of cells growing on solid surfaces with the *in vivo* conditions when the cells are embedded and exposed in a highly hydrated intercellular matrix. The cell-to-cell interactions may have an entirely different character.

## Conclusions

The mechanisms associated with maintenance of genome integrity of stem cells, particularly focused on elucidation of the role of ROS and DNA damage response, have been recently reviewed [153]. In this review, while we briefly outline most mechanisms, the emphasis is given to the role of HA, which was neglected in prior literature. Although most reviewed data comes from hematopoietic stem cells attempts have been made to summarize the evidence on a role of HA in other types of stem cells as well. Protection of genome of the germ cells (spermatozoa and oocytes) is even of more importance that of the stem cells. Since there is a significant association between HA and expression of HA receptors and genome integrity in spermatozoa and oocytes we draw therefore attention to the possible role of HA in protection of DNA integrity in germ cells as well, which was also uncared for in prior reviews.

As yet there is no direct experimental evidence that unequivocally demonstrates that HA protects stem cells from oxidative DNA damage. However, the wealth of indirect evidence presented in this review strongly advances the notion that one of the functions of HA associated with stem cells is DNA protection against oxidative damage. The most supporting evidence to this conception provide the findings that DNA damage response triggered by exogenous as well endogenous oxidants in several cell types, most likely reporting persistent oxidative damage, is strongly attenuated by HA [111, 113]. There is no rationale to expect that stem cells would be an exception and that DNA protection by this mechanism is not attainable in them. Since stem cells reside in the HA-rich niche, synthesize HA, express it on the surface and are able to internalize it [55-59], clearly conditions exist that allow HA to provide protection of DNA against oxidants.

Of interest and of further support for this notion are observations that the level of expression of HA on surface of stem cells correlates with their differentiation status and the mechanistic studies showing that HA modulates cell proclivity to differentiation and proliferation [55-61]. On the other hand, there are observations that oxygen tension gradient within the stem cell niche appears to provide signaling that mediates transition of these cells from quiescence to proliferation and trigger their differentiation [38-42, 154-156). It is tempting therefore to speculate that the oxygen tension gradient which mediates propensity of stem cells to differentiate is actually being modulated by the gradient of HA associated with stem cells. Thus, HA could have diverse functions with respect to stem cells. Specifically it can: (i) protect the most primitive long-term self-renewal stem cells from reactive oxidants; (ii) offer a gradient of accessibility of ROS which trigger their differentiation. This is achieved by different expression of HA-receptors and thus different level of HA binding and possibly its internalization, (iii) modulate motility of stem cells and progenitors [65, 70, 157], and (iv) similar to another glycosaminoglycan, heparin sulfate [158, 159], it may modulate gradients and accessibility to growth factors, and thus to control autocrine and paracrine signals for stem cells [158].
